# HaptGlove—Untethered Pneumatic Glove for Multimode Haptic Feedback in Reality–Virtuality Continuum

**DOI:** 10.1002/advs.202301044

**Published:** 2023-06-29

**Authors:** Jiaming Qi, Feng Gao, Guanghui Sun, Joo Chuan Yeo, Chwee Teck Lim

**Affiliations:** ^1^ Department of Biomedical Engineering National University of Singapore Singapore 117583 Singapore; ^2^ Institute for Health Innovation and Technology (iHealthtech) National University of Singapore Singapore 117599 Singapore; ^3^ School of Astronautics Harbin Institute of Technology Harbin 150001 China; ^4^ Mechanobiology Institute National University of Singapore Singapore 117411 Singapore; ^5^ SIA‐NUS Digital Aviation Corporate Lab National University of Singapore Singapore 117602 Singapore

**Keywords:** cutaneous feedback, Haptic glove, human‐computer interaction, kinesthetic feedback, multimode feedback, pneumatic

## Abstract

Novel haptics technologies are urgently needed to bridge the gap between entirely physical world and fully digital environment to render a more realistic and immersive human–computer interaction. Current virtual reality (VR) haptic gloves either deliver limited haptic feedback or are bulky and heavy. The authors develop a haptic glove or HaptGlove, an untethered and lightweight pneumatic glove, that allows users to “physically” interact in a VR environment and enables both kinesthetic and cutaneous sensations naturally and realistically. Integrated with five pairs of haptic feedback modules and fiber sensors, HaptGlove provides variable stiffness force feedback and fingertip force and vibration feedback, allowing users to touch, press, grasp, squeeze, and pull various virtual objects and feel the dynamic haptic changes. Significant improvements in VR realism and immersion are observed in a user study with participants achieving 78.9% accuracy in sorting six virtual balls of different stiffnesses. Importantly, HaptGlove facilitates VR training, education, entertainment, and socialization in a reality–virtuality continuum.

## Graphical abstract

An untethered and lightweight pneumatic haptic glove is developed to enable realistic touch sensation in the reality–virtuality continuum. Using soft pneumatic actuators and stretchable fiber sensors, the glove renders both kinesthetic and cutaneous feedback with imperceptible delay. Significantly improved realism and user immersion are observed in user studies, demonstrating the viability in virtual training, education, and entertainment.

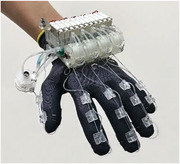

## 1 Introduction

Reality–virtuality continuum is a concept initially proposed by Milgram and Kishino in 1994, which frames immersive technologies, such as virtual reality (VR) or augmented reality (AR), into a continuum from entirely physical world to fully virtual environments.^[^ ^1^ ^]^ Providing realistic exteroception (sight, smell, hearing, touch, and taste) in a simulated virtual environment is essential for an immersive virtual experience that is coherent to the real‐world experience.^[^ ^2^ ^]^ However, one major limitation of current VR is the lack of realistic haptic feedback, which is critical to bring VR immersion to the next level.^[^ ^3^, ^4^ ^]^

Kinesthetic and cutaneous perceptions are two types of touch sensations received by mechanoreceptors in response to mechanical stimuli,^[^ ^5^, ^6^, ^7^, ^8^ ^]^ allowing us to feel the shape, softness, texture and weight of objects and manipulate them stably and precisely in daily life. Delivering haptics with more modalities has proven to improve immersion and enjoyment and facilitate training in VR.^[^ ^9^, ^10^ ^]^ However, it remains challenging to develop lightweight VR haptic devices in an untethered form factor that provides multimodal haptic feedback, including both kinesthetic and cutaneous feedback.

Electromagnetic motors are the most commonly used actuators in haptic devices because of their control flexibility and fast response, providing vibration,^[^ ^11^, ^12^ ^]^ fingertip directional forces,^[^ ^13^, ^14^, ^15^ ^]^ passive kinesthetic feedback^[^ ^16^, ^17^, ^18^ ^]^ and active kinesthetic feedback.^[^ ^19^, ^20^, ^21^, ^22^ ^]^ Skin‐integrated vibrotactile array with miniature vibration motors was developed for soft haptic interfaces.^[^ ^11^, ^12^ ^]^ For directional forces, parallel structures with small servo motors were used to deliver translational or rotational movement against the fingertips, allowing users to touch virtual objects and feel the object's weight, inertia, or stiffness.^[^ ^13^, ^14^, ^15^ ^]^ Kinesthetic haptic devices are classified into passive and active devices, depending on whether active force or motion is applied to user's fingers.^[^ ^3^ ^]^ For passive actuation, motors were used to trigger braking mechanisms to restrict finger motion. In this case, motors are lightweight and small because the resistive force is not directly generated from motors. For example, Wolverine^[^ ^18^ ^]^ and Dexmo^[^ ^16^ ^]^ implemented binary brakes triggered by lightweight motors to simulate rigid object interaction. Unlike passive actuation, devices with active actuation must consider the tradeoff between backdrivability and maximum torque.^[^ ^3^ ^]^ Hexotrac^[^ ^21^ ^]^ and WeHAPTIC^[^ ^19^ ^]^ delivered active kinesthetic feedback on fingertips with controllable force. However, the maximum resistive forces are usually lower because of the tradeoff on maximum motor torque.

Another widely used actuation type is electric field which includes piezoelectric actuation, dielectric elastomer (DE) actuation, and electrostatic (ES) actuation. Piezoelectric actuators have been implemented in a smart glove for vibration feedback.^[^ ^23^ ^]^ DE actuators were implemented to provide cutaneous feedback, such as vibration^[^ ^24^ ^]^ and fingertip force feedback.^[^ ^25^ ^]^ DE actuator with a spring roll shape was fabricated to provide active kinesthetic feedback on the palm side but with only a 5 mm range of motion.^[^ ^26^ ^]^ Using electrostatic adhesive, thin and lightweight cutaneous haptic actuators were fabricated, providing normal force, lateral force, and vibration on fingertips.^[^ ^27^ ^]^ Using similar mechanism, DextrES^[^ ^28^, ^29^ ^]^ was developed providing passive kinesthetic feedback. The maximum resistance varied according to the actuation voltage and the overlapping area. To better render softness sensation, a closed‐loop control scheme was proposed and evaluated,^[^ ^30^ ^]^ providing tunable force that recreated the grasping perception of nonlinear soft objects. Acting like a capacitance, actuation using electric fields is energy efficient. Although these actuators can be fabricated in a small, thin and lightweight form, DE and ES actuation usually require a high voltage up to hundreds or even thousands of volts, making the control system bulky and expensive. In addition, ES clutches only confer unidirectional resistance but cannot provide recovery force, reducing its realism of interacting with elastic virtual objects.

Pneumatic actuation is also widely used on wearable haptic devices. Most pneumatic actuators for cutaneous feedback are customized soft pneumatic actuators (SPA) with a deformable membrane which expands or contracts according to air inflation or deflation, providing vibration^[^ ^31^ ^]^ and fingertip force feedback.^[^ ^32^ ^]^ By designing a dense SPA array, higher resolution cutaneous feedback was delivered.^[^ ^33^ ^]^ Usually, the air pressure required for cutaneous feedback is not high (below 100 kPa). For kinesthetic feedback, air cylinders were implemented. For example, the Rutgers Master II equipped air cylinders at the palm side for active force feedback, but with limited finger range of motion.^[^ ^34^ ^]^ Air cylinders were also placed on the dorsal side of the hand, providing full finger flexion and extension.^[^ ^35^ ^]^ The actuation pressure for kinesthetic feedback usually achieves hundreds of kPa, therefore requiring a bulky and heavy pneumatic pump. Although pneumatic actuators are soft, lightweight, energy‐efficient, and provide high feedback force, the key obstacle for an untethered pneumatic glove is the size and weight of the pneumatic control system, which can be reduced by first designing pneumatic actuators requiring low actuation pressure and second combining simultaneously actuated channels into one.

**Figure 1:**
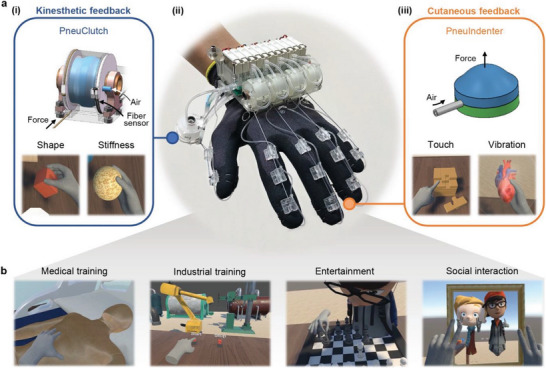
HaptGlove overview. a) (i)The kinesthetic feedback module, PneuClutch, delivering the shape and stiffness information of virtual objects. (ii) Image of HaptGlove worn on hand. (iii) The cutaneous feedback module, PneuIndenter, providing touch and vibration feedback. b) HaptGlove enhance virtual medical training, industrial training, entertainment and socialization.

Here, we report our haptic glove (called HaptGlove), a lightweight pneumatic glove that provides both kinesthetic and cutaneous feedback to the five fingers of a user in a fully untethered and wearable form factor (**Figure** **1**). The key novelty and innovation of our HaptGlove is the design, implementation and control of our low‐pressure actuated haptic feedback modules, PneuClutch and PneuIndenter. These modules enable HaptGlove to be untethered and lightweight, allowing users to freely explore the virtual world without any restrictions and being able to have a realistic sense of touch including shape, size, stiffness, and vibration in VR with imperceptible latency. VR scenarios were developed, from simple object grasping to a complex archery game, demonstrating our device's robustness and versatility. In addition, our user study indicated high accuracy for our HaptGlove to render different levels of stiffness in objects effectively and provide a more realistic and immersive VR experience. This presents numerous potential applications including in medical simulation, industrial training, entertainment, and social interaction in a virtual‐reality continuum.

## 2 Results

### 2.1 HaptGlove Design

Figure 1a(ii) illustrates HaptGlove that is portable and lightweight of 283 g, which is the lightest pneumatic haptic glove that provides both kinesthetic and cutaneous feedback (Table S1, Supporting Information). The control system is deployed on the back of the hand, comprising the pneumatic system, printed circuit board (PCB) and a lithium‐ion battery. A total of five PneuClutch modules are implemented next to the control system, providing kinesthetic feedback via cables connected to the glove digits. Similarly, five PneuIndenters are sewn on the glove fingertips with connecting air tubing to deliver cutaneous sensation. In addition, the embedded stretchable fiber sensors inside the PneuClutch modules can track finger bending. A Vive tracker is used per hand for palm tracking (Figure S1, Supporting Information). The workflow schematic of the HaptGlove is illustrated in Figure S2, Supporting Information.

### 2.2 PneuClutch Mechanism and Implementation

**Figure 2:**
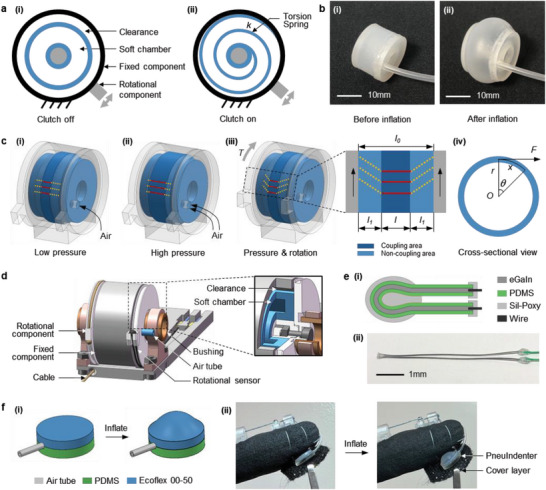
Mechanism and implementation of PneuClutch and PneuIndenter. a) Simplified cross‐sectional view of PneuClutch (i) before and (ii) after actuation. b) Images of soft chamber (i) before and (ii) after inflation. c) Illustrations of PneuClutch under (i) low pressure actuation, (ii) high pressure actuation, (iii) rotation after actuation and (iv) cross‐sectional view of (iii). Red lines and yellow dash lines indicates coupling and non‐coupling areas, respectively. d) PneuClutch implementation with an inset showing the inner structure. e) Rotational sensor (i) structure and (ii) image. f PneuIndenter (i) structure illustration and (ii) implementation beneath fingertip.

PneuClutch is a passive haptic module generating variable‐stiffness restrictive force on the fingertip by regulating actuation pressure. **Figure** **2**a(i) shows the cross‐sectional view of the simplified structure, revealing three concentric components, a fixed component, a rotational component, and a soft chamber. The soft chamber, with a hollow ring shape, is fixed on the rotational component. At normal state, the clearance between the soft chamber and the fixed component allows the rotational component to revolve freely. Upon pressurized, the soft chamber expands, overriding the clearance and coupling the rotational component with the fixed component (Figure 2a(ii)). In this state, the soft chamber acts as a torsion spring, providing restriction torque against rotation. Figure 2b(i,ii) show the soft chamber before and after inflation, respectively. The fabrication of the soft chamber is illustrated in Figure S3a, Supporting Information.

The coupling area becomes wider with increasing actuation pressure because of the further expansion of the soft chamber (Figure 2c(i,ii)). When rotation is applied after inflation (Figure 2c(iii)), the coupling area remains still, and the two side surfaces contacting the rotational component revolve at the same angle, which causes the shear stress in the non‐coupling area, contributing to the restriction torque. To calculate the feedback force *F* at a given rotation *θ*, we assume the non‐coupling area of the soft chamber is subjected to pure shear forces. First, we define *l*_0_, *l*, and *l*_1_ in Figure 2c(iii) as the width of the soft chamber, coupling area and non‐coupling area, respectively. From the side view shown in Figure 2c(iv), the torque *T* is described as *T = Fr = kθ*, where *r* is the radius of the soft chamber and *k* is the rotational stiffness of the coupled structure. The rotation angle is described as *θ = Tl*_1_/(2*GI*_p_), where *G* is the elastomer's shear modulus, *I*_p_ is the polar moment of inertia, and *GI*_p_ is the torsional rigidity. Therefore, we derive the feedback force *F* as Equation (1).

(1) $$F=\frac{4GI_{p}}{r\left(l_{0}-l\right)}\theta =\frac{4GI_{p}}{r^{2}\left(l_{0}-l\right)}x=\kappa x$$

where *x* is the arc length and *κ* is the equivalent linear stiffness. In our case, *l* is the only variable that changes *κ*. In addition, *l* is a function of actuation pressure *p* with a positive correlation. This indicates that PneuClutch can perform as a variable stiffness joint to provide feedback force by regulating the pressure inside the soft chamber.

Figure 2d and the inset show the implementation of PneuClutch and its inner structure, weighing only 14 g. A Dyneema cable is attached to the rotational component with another end connecting the fingertip to transmit the feedback force. To minimize rotational friction, a pair of composite copper bushings is used. The rotational sensor (Figure 2e) is a thin polydimethylsiloxane (PDMS) microtube injected with eutectic gallium indium (eGaIn) as the sensing element, whose resistance increases when stretched.^[^ ^36^, ^37^ ^]^ The sensor is highly flexible and stretchable, with a length of 11 mm and an outer diameter of 0.56 mm. For sensor implementation, the two ends are attached to the fixed component, and the middle is attached to the rotational component. This configuration allows the sensor be stretched or released during rotation resulting in electrical resistance changes. Because the cable is restricted to tensile forces, the elastic sensor provides an opposing force to pull the rotational component back to the initial position. Movie S1, Supporting Information demonstrates the PneuClutch force feedback with variable actuation pressure.

### 2.3 PneuIndenter Mechanism and Implementation

PneuIndenter is an active haptic module generating variable force and vibration feedback on fingertip via skin indentation. Each PneuIndenter has two circle‐shaped elastomer sheets which are glued together with an air tube (Figure 2f(i)). The top and bottom sheets have the same dimension with 9 mm in diameter and 1.5 mm in thickness but are made from Ecoflex 00–50 and PDMS, respectively. Both elastomer layers were fabricated through a casting process (Figure S3b, Supporting Information). Because of the differences in Young's moduli between Ecoflex and PDMS, expansion occurs on the top layer when inflated, which causes the indentation direction to be normal to the finger pad. By regulating the actuation pressure and frequency, skin indentation can be controlled, generating controllable force and vibration to fingertip. Shown in Figure 2f(ii), PneuIndenter is placed beneath the fingertip and covered with a thin layer of fabric sewn to the glove. An air tube connects the PneuIndenter to the pneumatic system. Movie S2, Supporting Information demonstrates the activation of PneuIndenter with variable pressure and frequency.

### 2.4 Control System

**Figure 3:**
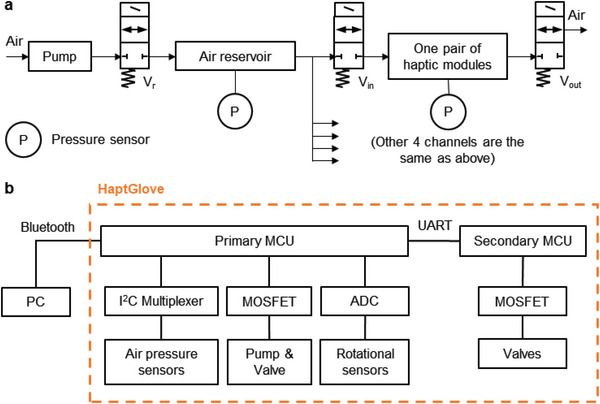
HaptGlove control system. a) Schematics of pneumatic system. Because all five pairs of haptic modules in the pneumatic system uses the same control method, only one is illustrated in the schematic for simplicity. b) Schematics of electrical system.

The pneumatic system comprises 11 solenoid valves (S070B‐RAG‐X50), a mini pump (DQB020‐A), a 3D‐printed manifold and six air pressure sensors (MS5637‐02BA03). **Figure** **3**a shows the schematic of the pneumatic system. One pump, one solenoid valve (*V*_r_) and one air pressure sensor are used to maintain stable pressure in the air reservoir. Importantly, one pair of haptic modules for each finger shares the same air pathway and will be inflated or deflated simultaneously with the same actuation pressure. Haptic modules are activated by precisely controlling the on and off timings of valves *V*_in_ and *V*_out_ in microseconds. The opening intervals of *V*_in_ and *V*_out_ valves in each pneumatic channel are precalibrated to achieve precise pressure control for delivering accurate and consistent haptic feedback.

In addition, the haptic feedback is energy efficient. *V*_in_ and *V*_out_ valves act only when the haptic information changes, for example, on the time points of grasping or releasing an object. When there are no changes in haptics, for example, holding the same virtual object, *V*_in_ and *V*_out_ valves stay closed, keeping the pressurized air in haptic modules, and maintaining the haptic feedback without consuming any power. We compared the power consumption between our HaptGlove system with five off‐the‐shelf eccentric rotating mass (ERM) motors in a grasping and holding virtual ball experiment (Figure S4, Supporting Information). The average power of HaptGlove was 1.12 W in this grasping event which is 54.9% lesser than using ERM motors (2.43 W).

Figure 3b shows the schematic of the electrical system. The primary MCU reads the data at 50 Hz from six air pressure sensors using *I* ^2^ *C* bus and five rotational sensors using ADC for finger tracking. Data is transmitted to the computer via Bluetooth. The primary MCU also controls the pump and *V*_r_ valve to maintain the air pressure stability in the reservoir. After receiving the information regarding the haptic interaction from the computer, the primary MCU passes the received data to the secondary MCU via UART communication. Then, the secondary MCU decodes the data and precisely controls the on and off timing of the corresponding *V*_in_ and *V*_out_ valves.

### 2.5 Performance Evaluation

**Figure 4:**
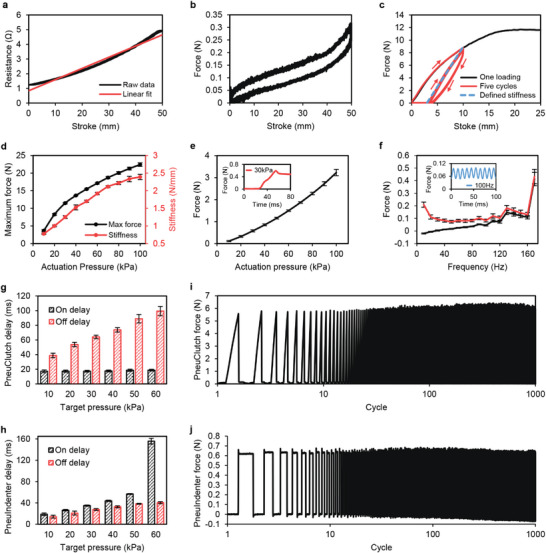
Performance evaluation of haptic feedback modules. a) Rotational sensor (implemented on PneuClutch) resistance relationship with stroke. b) PneuClutch free movement resistive force. c) PneuClutch force feedback profile under 30 kPa actuation. d) PneuClutch maximum resistive force and variable stiffness range with 10 to 100 kPa actuation. e) PneuIndenter variable fingertip force with 10 to 100 kPa actuation. Inset shows the feedback force curve under 30 kPa actuation. f) PneuIndenter vibration feedback with actuation frequencies from 10 to 170 Hz. Inset shows the feedback force curve under 100 Hz actuation. g) PneuClutch delay for generating and releasing haptic feedback, noted as on delay and off delay, respectively. h) PneuIndenter delay for generating and releasing haptic feedback, noted as on delay and off delay, respectively. i,j) Force feedback consistency of PneuClutch and PneuIndenter with 1000‐cycle testing.

Testing set up for PneuClutch and PneuIndenter are illustrated in Figure S5a,b, Supporting Information, respectively. The pneumatic system is used for actuation, providing accurate and consistent pressure for haptic feedback modules (Figure S6, Supporting Information). When PneuClutch is pulled, the electrical resistance profile of the rotation sensor indicates a positive correlation with the stroke, ranging from 1.24 to 4.92 Ω with negligible hysteresis and good linearity, indicating a stable rotation sensing capability (**Figure** **4**a). When haptics is not applied, the resistive force (Figure 4b) is owing to the elasticity of the rotational sensor. Because the sensor is ultrathin and highly stretchable, the resistive force changes smoothly with a maximum value of 0.32 N, which is nearly unperceivable.

Next, we tested PneuClutch variable stiffness force feedback performance. Figure 4c shows an example of the force curves under 30 kPa actuation. Under a 25 mm stroke, the resistive force increases and achieves a maximum value of 11.7 N. The nonlinearity in the feedback force curve is because the edge of the contact area gradually overcomes the friction and slides along the rotation direction as the rotation angle increases. The sliding decreases the rendered stiffness. Therefore, the slope of the force curve gradually becomes flat. When the feedback force is high enough to reach the maximum friction between the soft chamber and the fixed component, the soft chamber slides with the rotational component along the inner surface, generating a constant frictional force. Under the five cycles of loading with a 10 mm stroke, a significant hysteresis was observed in the first cycle because of the sliding, while the force profile in the rest of the loadings became consistent with a smaller hysteresis indicating no further sliding was observed in the subsequent cycles. This smaller hysteresis is because of the mechanical hysteresis of the silicone rubber material used for fabricating the soft chamber. Here, we define the force feedback stiffness as the slope of the linear fit line based on the force data during loading after the first cycle, which is 1.25 N mm^−1^ under 30 kPa actuation pressure. We further tested PneuClutch with an actuation pressure range from 10 to 100 kPa with 10 kPa increment (Figure 4d). The maximum resistive force increases from 3.65 to 22.41 N with increasing actuation pressure. The force feedback stiffness ranges from 0.78 to 2.39N mm^−1^, indicating a variable stiffness capability by regulating actuation pressure. The error bars are the standard deviation based on three PneuClutch modules, and each module performed three repetitive testings. Consistent performance among different samples demonstrates reliable force feedback to users’ multiple fingers.

By increasing the actuation pressure from 10 to 100 kPa, the indentation force increases from 0.11 to 3.23 N, providing the variable fingertip force feedback (Figure 4e). The inset shows the measured force curve under 30 kPa actuation. PneuIndenter has a fast response to the change of actuation pressure. Figure 4f shows the peak and valley forces of PneuIndenter vibration under different actuation frequencies from 10 to 170 Hz. It is observed that the actuation magnitude becomes smaller with increasing actuation frequency. This is due to the air resistance that the reduced air is delivered in and exhausted from PneuIndenter within a shorter actuation cycle. When the actuation frequency is above 160 Hz, insufficient time is given for the air to be exhausted in the PneuIndenter. A much larger error bar at 170 Hz indicates that the vibration is less reliable. Therefore, PneuIndenter can perform stable vibration feedback up to 160 Hz. The inset shows a consistent vibrating force curve under 100 Hz actuation.

Visual‐haptics delay is one of the critical parameters for VR haptic devices. Figure 4g,h illustrate the measured haptics generation and removal delay (noted as haptics on and off delay). In general, haptics on delay for PneuClutch is shorter than PneuIndenter because the definitions of generating haptics delay for passive and active modules are different (Figure S7, Supporting Information), and PneuClutch is closer to the air reservoir with smaller air resistance. But haptics off delay for PneuIndenter is shorter because the indentation force accelerates the air exhaustion. For PneuClutch, it only takes around 18 to be activated regardless of target pressure. But it takes longer to free the clutch and the time required increases from 38.8 to 99.4 ms with an increasing actuation pressure. For PneuIndenter, as the target pressure increases, the haptics on delay increases from 18.8 to 155.9 ms and the haptics off delay also increases from 14.1 to 40.3 ms.

To test the haptic feedback consistency, we performed 1000‐cycle repetitive testing with the same actuation pressure. Consistent feedback forces are shown in Figure 4i,j, indicating the long‐term reliability of our modules for both kinesthetic and cutaneous sensations. A detailed comparison of key performances between our HaptGlove and the current state‐of‐the‐art haptic glove prototypes and commercial products can be found in Table S1, Supporting Information. These include kinesthetic and cutaneous feedback capabilities, finger tracking, weight and portability.

### 2.6 VR Applications Development

**Figure 5:**
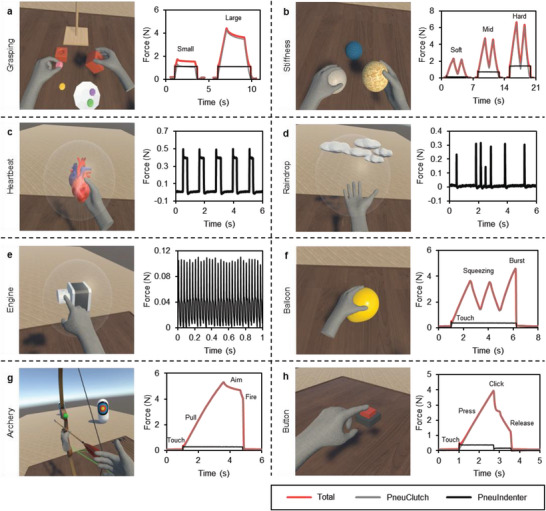
VR applications and corresponding feedback forces. a) Grasping large and small objects. b) Squeezing balls with different stiffness. c–e) Feeling of heartbeat, raindrops and engine vibration. f) Squeezing and bursting a balloon. g) Archery simulator. h) Pressing a button.

Several application scenarios were developed in Unity3D to demonstrate the multimode haptic feedback abilities of HaptGlove. Finger calibration (Figure S8, Supporting Information) was done for accurate and consistent finger tracking among different users. **Figure** **5** shows the applications and the corresponding feedback force on one finger from two haptic feedback modules. For a more accurate measurement, forces from PneuClutch and PneuIndenter were measured separately using mechanical tester with the same testing setup shown in Figure S4, Supporting Information, where the mechanical tester was to mimic the finger movement. For VR scenarios where both haptic modules were needed, the joint forces were calculated using the root sum square of two forces as the force directions were assumed perpendicular based on the configuration illustrated in Figure S9, Supporting Information.

Figure 5a and Movie S3, Supporting Information demonstrate the grasping of large and small objects. Briefly, users grasped virtual objects and manipulated them precisely in VR using HaptGlove. In this scenario, all objects were designed to be fairly rigid with 50 kPa actuation. Contact sensation is felt as the hand avatar touches the virtual object, which is the cutaneous sensation provided by PneuIndenter. When a user grasps and holds the object, kinesthetic feedback force is felt from PneuClutch and fingertips are restricted at the object's surface. When touching objects with different stiffness, haptic feedback modules are actuated accordingly, which allows users to distinguish between soft and hard objects. Figure 5b and Movie S4, Supporting Information show manipulation of three elastic balls with different stiffnesses, which is achieved using three actuation pressures, 10, 35, and 55 kPa. The force curves show, first, soft ball provides softer cutaneous feedback. Second, soft ball is easier to squeeze with a smaller resistive force. In addition, a smooth increasing and decreasing force according to user squeezing creates a realistic touch sensation of elastic objects. By controlling the actuation frequency to 0.83 Hz with 20 kPa pressure, our HaptGlove renders the cutaneous sensation of a beating heart (Figure 5c). Figure 5d shows users feeling the raindrops on their fingertips. This sensation was achieved by randomly activating the haptic modules for 100 ms with a random actuation pressure from 10 to 20 kPa at 3 Hz. In Figure 5e, we controlled the actuation frequency of the haptic module for the index finger at 30 Hz to mimic the sensation when touching a vibrating motor. Movie S5, Supporting Information demonstrates the sensing of heartbeat, raindrops and engine vibration. In Figure 5f, we created an elastic balloon with 20 kPa actuation. Users feel the contact and elasticity when they touch and squeeze the balloon. When the user squeezes hard, the balloon explodes and applied haptics are released simultaneously, creating a realistic bursting sensation (Movie S6, Supporting Information). Using HaptGlove, we also designed an archery game (Figure 5g and Movie S7, Supporting Information) that allowed users to feel the string tension when shooting arrows. Users first feel their index, middle and ring fingers touch the string. Then, they feel the increasing tension when they flex fingers while pulling the string. After aiming at the target, a small finger extension fires the arrow, removing applied haptics simultaneously. We also created a realistic virtual button by dynamically changing the actuation pressure according to users’ pressing (Figure 5h and Movie S6, Supporting Information). When the index finger touches the button, the haptic feedback modules are activated with 25 kPa, so that users feel the contact and the button resistance when pressing it by flexing the index finger. At the point when the button is pressed, half of the air inside the haptic feedback modules is exhausted, causing a sudden drop of both kinesthetic and cutaneous feedback, which mimics the click sensation. Then, as the user continuously extends the index finger to release the button, a corresponding decreasing force is felt until the finger removes from the button.

### 2.7 User Study

User studies were conducted to evaluate if HaptGlove can provide multimode haptic feedback effectively and if the provided haptic feedback improves the VR user experience. 19 participants (11 male and 8 female) were recruited, with age ranging from 21 to 41. All user experiments were approved by the Institutional Review Board, National University of Singapore (NUS‐IRB‐2022‐70) and were carried out with the full, informed consent of the subjects.

We prepared two pairs of HaptGlove with different sizes. Participants were asked to put on one glove with a suitable size on their dominant hand. Then put on noise cancelling headphones playing white noise to block sound from the device and environment that may affect the haptics perception. Finally, a VR headset (HTC Vive Pro 2) was worn to display rendered VR world.

**Figure 6:**
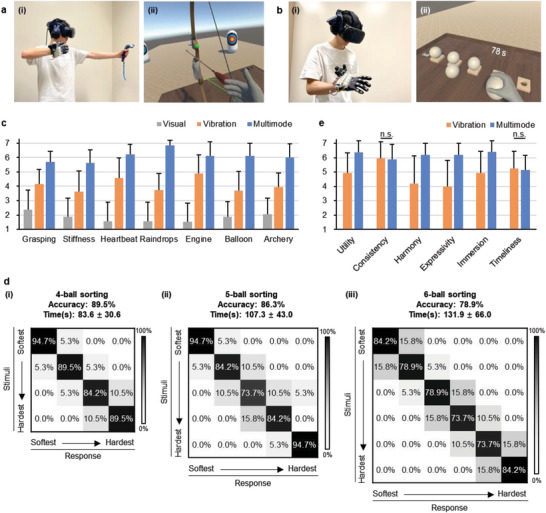
HaptGlove user study. a) (i) Image of a user playing archery game and his (ii) VR view. b) (i) Image of a user performing ball sorting experiment and his VR view. c) Realism rating results. d) Questionnaire rating results. e) Confusion matrix of (i) 4‐ball sorting, (ii) 5‐ball sorting and (iii) 6‐ball sorting.

There were two VR haptics experiments. The first experiment was to evaluate haptic feedback realism. Participants experienced seven scenarios (shown in Figure 5a–g) under three conditions, no haptics (visual), vibration motor haptics (vibration) and multimode haptics (multimode). For the Vibration condition, we attached off‐the‐shelf ERM motors to fingertips (Figure S10, Supporting Information) and controlled them using pulse width modulation (PWM). A 7‐point Likert scale was used, with 1 indicating not realistic and 7 indicating very realistic. **Figure** **6**a shows a user playing the archery game and his VR view.

The second experiment was to quantitatively evaluate the effectiveness of rendering soft and hard objects under the Multimode by performing virtual ball sorting. A set of visually identical virtual balls were presented on a virtual table (Figure S11, Supporting Information) with randomly assigned stiffness. The softest and hardest balls were equivalent to 10 and 60 kPa actuation, respectively. The actuation pressure for other balls was assigned with an equal difference. Participants continuously performed one 4‐ball, one 5‐ball, and one 6‐ball sorting. For each sorting, they were asked to finish within 2 min. Figure 6b and Movie S8, Supporting Information shows a user performing 6‐ball sorting experiment and his VR view.

Then, a modified questionnaire based on literature^[^ ^38^ ^]^ regarding the general evaluation of haptics experience was given, evaluating Utility (Is it useful?), Consistency (Is it reliable?), Harmony (Does it fit with visual sense?), Expressivity (Is there distinction between haptic objects?), Immersion (Do you feel engaged?) and Timeliness (How timely is the haptics delivery?). A 7‐point Likert scale was used with 1 indicating negative and 7 indicating positive. Also, a short discussion with the participant was ensued.

One‐way repeated measures ANOVA with Bonferroni correction were performed for analyzing realism rating results (Figure 6c). Significant differences are found in all tested VR scenarios, grasping (*F*_2,36_ = 62.8, *p* < 0.001), stiffness (*F*_2,36_ = 66.4, *p* < 0.001), heartbeat (*F*_2,36_ = 85.3, *p* < 0.001), raindrops (*F*_2,36_ = 146.6, *p* < 0.001), engine (*F*_2,36_ = 72.1, *p* < 0.001), balloon (*F*_2,36_ = 89.0, *p* < 0.001), and archery (*F*_2,36_ = 93.3, *p* < 0.001). In addition, the post‐hoc pairwise comparison shows that there is a significant difference between any two conditions in each VR scenario. In general, vibration feedback significantly improves the VR realism experience, but multimode haptics is significantly more realistic. In the grasping scene, the kinesthetic force feedback restricted participants’ fingers at the object's surface, causing much less penetration inside the virtual objects and making grasping more realistic (5.68, S.D. 0.75). In the stiffness sensing scenario, the variable stiffness capability in the multimode realistically recreated different elasticities of the objects (5.63, S.D. 0.90), while under the vibration mode, different stiffnesses were expressed by magnitude of vibration which lacked fidelity. For the heartbeat, most of the participants felt the multimode was more realistic (6.21, S.D. 0.71), but 10.5% of participants preferred vibration because ERM motors vibrated a much larger area which gave them an illusion that they were holding a big heart that vibrated as a whole. Raindrops scenario was rated as the most realistic for the multimode because of the sharp and localized cutaneous force feedback. The rating (6.87, S.D. 0.37) is 83.4% higher than vibration (3.47, S.D. 1.15). In addition, the Raindrops was the most popular scenario, with 47.3% of participants rated as their favorite because of the realistic feeling. For the engine scene, 68.4% of participants rated the multimode as more realistic (6.11, S.D. 0.99) because the 30 Hz force feedback made them feel the blade was continuously hitting their fingertip. In the balloon scene, the rating of the multimode (6.11, S.D. 0.88) is 66% higher than vibration mode (3.68, S.D. 1.34) because the kinesthetic feedback and fast response well recreate the balloon elasticity and the bursting. For the archery, the feeling of string tension and arrow release from kinesthetic feedback made this scene more realistic under the multimode (6, S.D. 0.94). In addition, the archery is the second most popular, with 26.3% of participants considering it as their favorite.

In the stiffness sorting experiment (Figure 6d), the average time required to finish a 4‐ball sorting is 83.6 s with an average accuracy of 89.5%. 79.9% of participants successfully sorted all 4 balls correctly. By increasing the number of balls to 5 and 6, the average accuracy drops by 3.2% and 7.4%, and the average time increases by 23.4 and 24.6 s. It is intuitive that participants tend to spend more time and make slightly more errors with a larger number of balls because the stiffness difference between two levels is smaller, but results still show a high average accuracy of 78.9% for 6‐ball sorting, indicating the capability to render multiple levels of stiffness for virtual objects.

Paired *t*‐test was performed for analyzing questionnaire results (Figure 6e). First, positive ratings with no significant difference were observed in consistency (*p* = 0.587) and timeliness (*p* = 0.675), indicating that our PneuClutch and PneuIndenter were reliable and fast‐actuated compared with ERM motors. Both haptic feedback conditions were rated as useful but the multimode (6.36, S.D. 0.83) was significantly more useful (*p* < 0.001) than the vibration (4.94, S.D. 1.39) because of the high fidelity provided. The vibration (4.21, S.D. 1.93) is significantly less harmonious with visual sense (*p* < 0.001) than the multimode (6.21, S.D. 0.78). Some participants commented that vibration was not aligned with their visual sense because they did not expect virtual balls to vibrate when they touched them. The multimode (6.21, S.D. 0.78) shows significantly more expressivity (*p* < 0.001) because PneuClutch and PneuIndenter created richer haptic feedback, generating virtual objects with distinctive haptics sensing. The multimode (6.42, S.D. 0.769) also provides a significantly more immersive VR experience (*p* < 0.001).

The above user study showed that HaptGlove provided consistent and timely multimode haptic feedback and delivered a much more realistic touch sensation, which significantly improves VR user experience. In addition, 78.9% of participants commented the weight was acceptable. Some commented that the weight was unnoticeable when immersed in VR.

## 3 Conclusion

To enhance human–computer interaction (HCI), haptic gloves allow users to naturally interact with VR, which poses a unique advantage compared with handheld controllers. We presented HaptGlove, the first untethered and lightweight pneumatic haptic glove that provides timely and realistic multimodal feedback, and has the following unique advantages:

a) Fully wireless with excellent portability: Our HaptGlove is fully wireless as our novel haptic feedback modules are compact and lightweight, and can generate rich haptic feedback with low air pressure. The pneumatic components can be miniaturized to accomplish this low pressure supply. Therefore, the whole control system can be deployed on the back of the hand, enabling it to be fully wireless with a weight of only 283 g which is much lighter than most available haptic gloves.
b) Realistic artificial haptics sensation with low power consumption. HaptGlove provides both kinesthetic and cutaneous feedback using our novel ultralight pneumatic haptic feedback modules, enabling the sensing of the shape and stiffness of virtual objects apart from being able to feel raindrops on the fingertips with low power consumption. HaptGlove also allows users to touch, press, grasp, squeeze, and pull various objects and feel the dynamic haptic changes. Most existing haptic gloves only provide either kinesthetic or cutaneous feedback, which limits the realism perception.
c) Imperceptible latency. HaptGlove delivers imperceptible visual‐haptics delay of as low as 20 ms because the wireless implementation enables the haptic feedback modules to be located next to the pneumatic system, significantly reducing the flow resistance. In addition, the low pressure required for haptic feedback reduces the amount of air needed, contributing to the timely response as well. This imperceptible latency allows HaptGlove to respond quickly according to users’ real‐time actions, creating an immersive and realistic interaction.

Despite the hysteresis observed in PneuClutch, the feedback force smoothly decreases during finger extension after the squeezing, recreating a realistic feeling of squeezing elastic objects. In addition, as a tradeoff between portability and high‐resolution cutaneous feedback, HaptGlove has one PneuIndenter for each finger, but it is proven practical to convey rich haptic effects. The lightweight mini pump brings good portability but limits the maximum actuation pressure to 60 kPa. Incorporating a more powerful pump could further improve the HaptGlove performance. Although different glove sizes were prepared for different users in the user study for consistent haptic feedback, we did not precisely control the initial static pressure on the fingertip, which may vary slightly from person to person and thus, may slightly affect the sensations. HaptGlove has demonstrated enhanced user experience in touching, pressing, grasping and pulling various virtual objects in response to dynamic haptic changes. Importantly, the user study reveals improved realism and immersion compared to conventional vibrational feedback, demonstrating the effectiveness of HaptGlove.

Beyond single‐person interaction with virtual objects, social interactions with multiple users were achieved. Here, a two‐user chess game demonstrates scenarios for multiple users to pick up and feel chess pieces and even feel physical contact with each other through shaking hands and doing high‐fives (Movie S9, Supporting Information). HaptGlove adds an additional layer of sense of touch that enhances social interaction in the emerging metaverse.

Additionally, realistic haptic feedback not only improves immersion but also delivers information from another dimension, making VR content more informative. For example, in medical education, meaningful information can be conveyed through touch, such as via palpation. HaptGlove has the potential to be implemented to enable “physical” touching of virtual patients and feeling of tissue stiffness during VR medical training. This facilitates a decentralized VR medical training platform where trainees can perform realistic training programs remotely and communicate with instructors in real‐time. HaptGlove can also be used for VR industrial training and simulation, where buttons and tools can be felt with haptic feedback for an effective learning and training experience. With HaptGlove, hand rehabilitation can also be done to train patients’ hand–eye coordination and improve finger strength and range of motion. With gamified training content, patients can be more motivated to practice. In general, HaptGlove proposes an untethered and lightweight solution for a natural HCI with realistic haptic feedback, which facilitates VR training, education, entertainment and social interactions in reality–virtuality continuum.

## 4 Experimental Section

### Fabrication of Soft Pneumatic Actuators

In Figure S3, Supporting Information, molds were 3D‐printed with Form 3 printer using Clear Resin. To fabricate the soft chamber in PneuClutch, a mixture of Ecoflex 00–50 with 1:1 weight ratio of part A and B was poured inside the molds. Similarly, Ecoflex mixture was poured into one mold to create the top layer of PneuIndenter. Next, a mixture of PDMS (Sylgard 184, Dow Corning) with 10:1 weight ratio of base elastomer and curing agent was poured into another mold to create the bottom layer of PneuIndenter. The silicone elastomers were cured in a 70 °C oven overnight. Then, cured elastomers were demolded and glued using Sil‐Poxy.

### Experimental Setup

PneuClutch was clamped to a mechanical tester (Shimadzu EZ‐SX), while the cable was connected to the force sensor (SM‐500N‐168) on the moving slider of the mechanical tester for force measurement. The rotational sensor was connected to the digital multimeter (Keithley DMM6500) for resistance measurement. PneuIndenter was placed right beneath a flat indenter on the mechanical tester. When air was pumped inside the PneuIndenter, the top Ecoflex layer expanded, and the force sensor recorded the corresponding force. In the power consumption measurement, a DC power supply (Keithley 2220‐30‐1) was used to power the HaptGlove at 3.7 V, and the digital multimeter was connected in series to continuously measure the current value.

### Hand Tracking and Calibration

Palm and finger tracking were performed to map hand avatars. One HTC Vive Tracker was used for each hand to obtain the palm position and orientation in six degrees of freedom. Electrical resistance data from five rotational sensors were read continuously for finger tracking of each hand. A three‐step finger calibration method was adopted by doing “Thumbs Up,” “Four,” and “Pinch” poses (Figure S8, Supporting Information). The respective values of the five rotational sensors from the poses were used to map the finger positions.

## Conflict of Interest

J.Q. and C.T.L. are inventors on a pending patent related to this work filed by the National University of Singapore (no. 10202205047P, filed on 13 May 2022). The authors declare no other competing interests.

## Author Contributions

C.T.L., J.C.Y., and J.Q. conceived the idea. J.Q. and J.C.Y. designed the experiments. F.G. and J.Q. completed the circuit design. J.Q. completed the mechanical design, wrote control code, and conducted the experiments. J.Q. drafted the manuscript. C.T.L., J.C.Y., J.Q., F.G., and G.S. edited the manuscript.

## Supporting information

Supporting Information

Supplemental Movie 1

Supplemental Movie 2

Supplemental Movie 3

Supplemental Movie 4

Supplemental Movie 5

Supplemental Movie 6

Supplemental Movie 7

Supplemental Movie 8

Supplemental Movie 9

## Data Availability

The data that support the findings of this study are available in the supplementary material of this article.
