# Supplementary material for: HaptGlove—Untethered Pneumatic Glove for Multimode Haptic Feedback in Reality–Virtuality Continuum
Source: Adv Sci (Weinh). 2023 Jun 29;10(25):2301044. doi: 10.1002/advs.202301044 (PMC10477838; doi:10.1002/advs.202301044)
Supplement: Supplementary file 1 — Supporting Information [file ADVS-10-2301044-s002.pdf]

ADVANCED SCIENCE
Open Access

Supporting Information

for *Adv. Sci.*, DOI 10.1002/advs.202301044

HaptGlove—Untethered Pneumatic Glove for Multimode Haptic Feedback in Reality–Virtuality Continuum

Jiaming Qi, Feng Gao, Guanghui Sun, Joo Chuan Yeo and Chwee Teck Lim**

Supporting Information

HaptGlove - Untethered pneumatic glove for multimode haptic feedback in reality-virtuality continuum

Jiaming Qi, Feng Gao, Guanghui Sun, Joo Chuan Yeo, Chwee Teck Lim**

This PDF file includes:

Figure S1 to S11
Table S1
Movie S1 to S9

Other Supplementary Materials for this manuscript include the following:

Movie S1 to S9

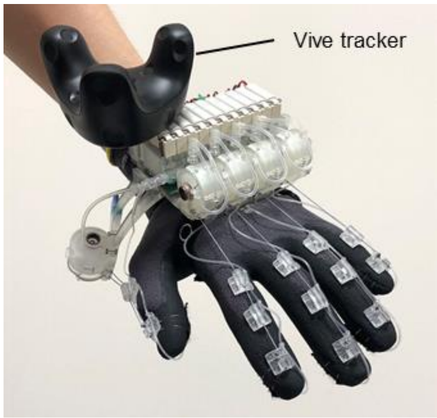

Figure S1. HaptGlove with Vive Tracker.

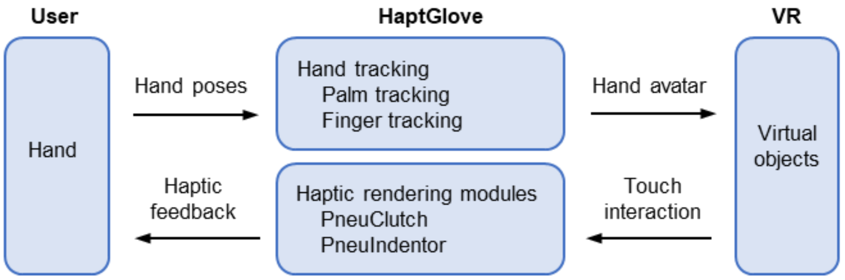

Figure S2. Workflow schematic of HaptGlove, indicating the interactions between virtual objects and corresponding haptic responses on the user's hand. Briefly, the HaptGlove monitors user's hand poses by palm and finger tracking using Vive tracker and rotational sensors, respectively. The information is mapped onto a hand avatar in VR. Information regarding touch interactions with virtual objects are sent back to HaptGlove to process, and the corresponding haptic rendering modules are activated to provide haptic feedback to the user.

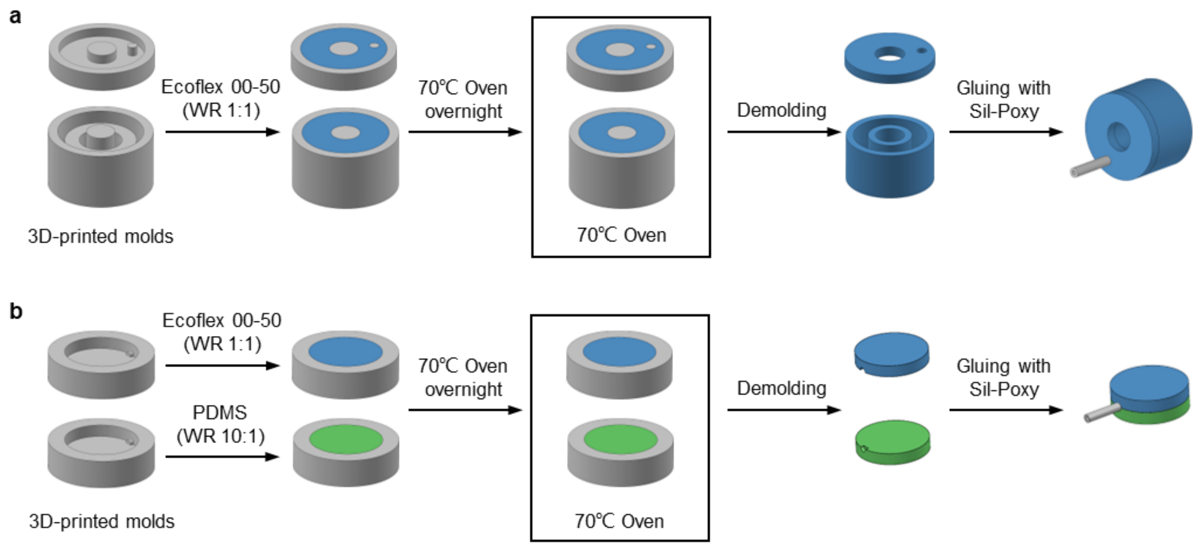

Figure S3. Soft actuator fabrication. (a) PneuClutch soft chamber fabrication. (b) PneuIndenter fabrication. WR stands for weight ratio.

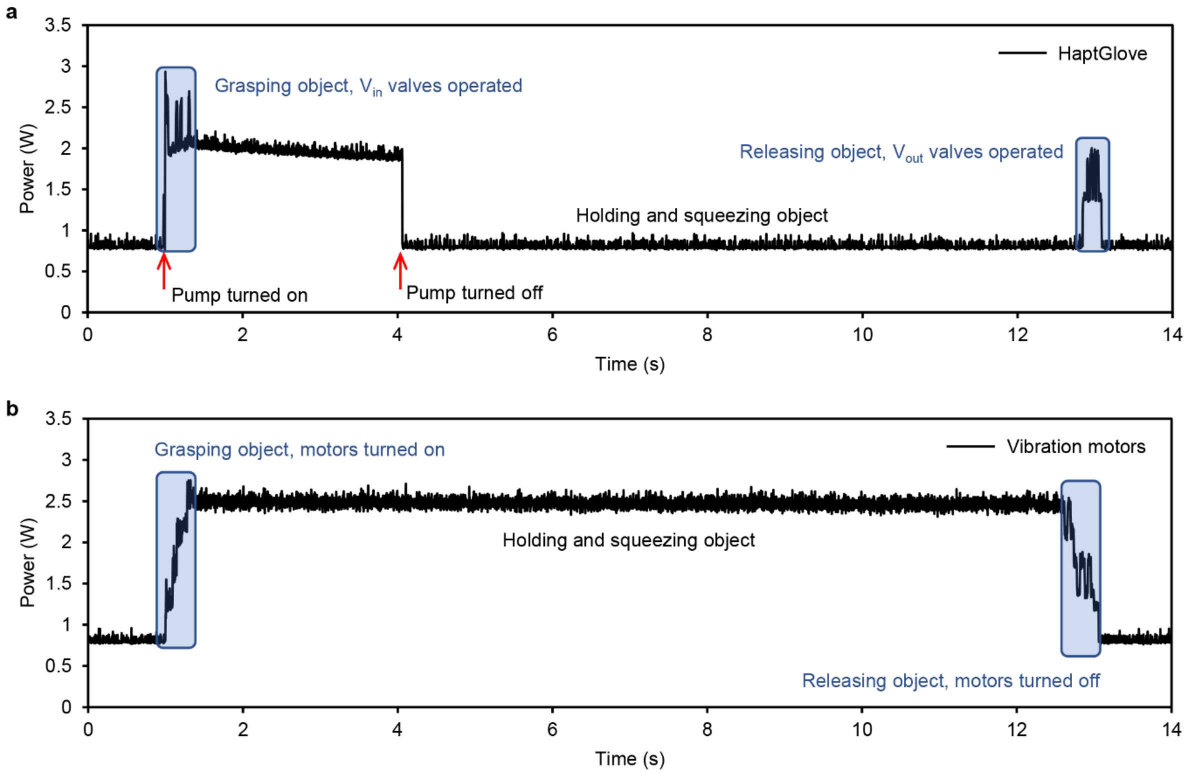

Figure S4. HaptGlove power consumption of grasping a virtual ball. (a) Multimode haptic feedback. When fingers touched virtual objects, corresponding valves operated to actuate the PneuClutches and PneuIndenters. At the same time, the pump was turned on to maintain the pressure in the reservoir. After these operations were done, the virtual ball can still be constantly felt in hand while the system power consumption was kept at a low level. The average power in this grasping event was 1.12 W. (b) Vibration haptic feedback using eccentric rotating mass (ERM) motors. The ERM motors need to be kept on to create a continuous grasping sensation. The average power was 2.43 W.

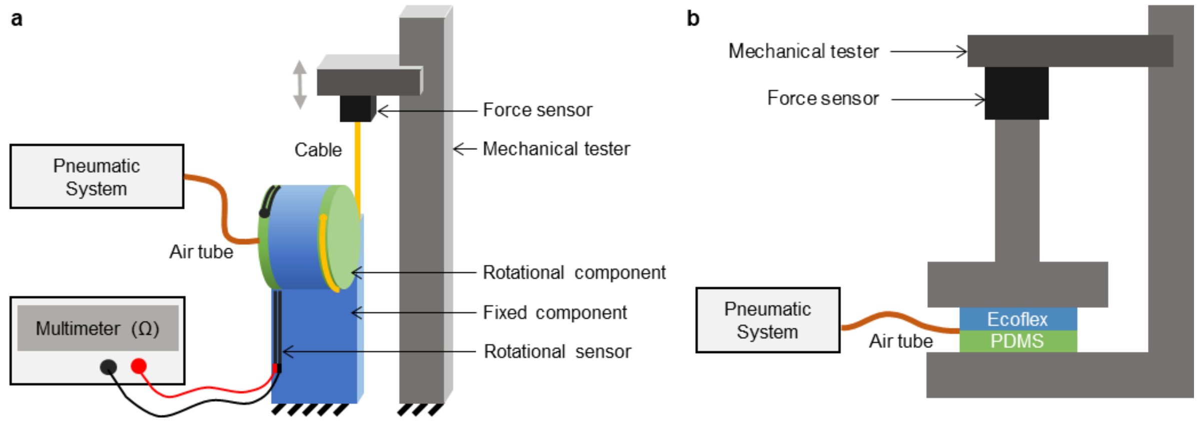

Figure S5. Testing setup of (a) PneuClutch and (b) PneuIndenter.

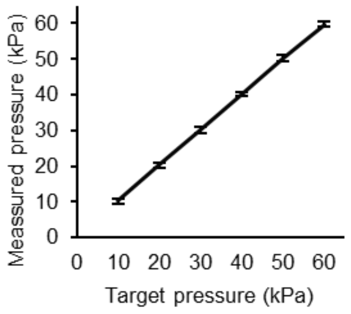

Figure S6. Pneumatic system pressure delivery test.

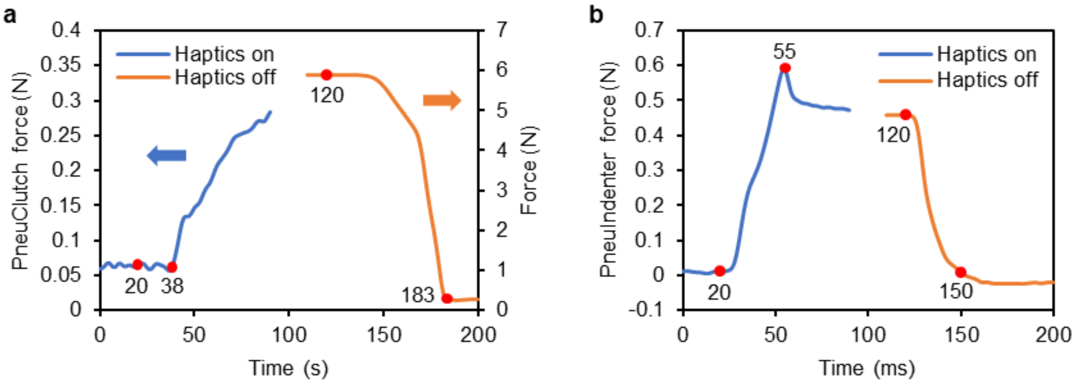

Figure S7. Force curves with 30kPa actuation showing the on and off delay of PneuClutch and PneuIndenter. For haptics on delay, because PneuClutch is a passive module, the delay starts when the V_{in} valve is powered and ends when the feedback force starts to increase. While PneuIndenter is an active device, the haptics on delay ends at the time when the force reached 95% of its peak valve. The haptics off delay for both modules is between the time when V_{out} valve is powered to the time when the feedback force drops below 5%. (a) 18ms and 63ms were observed for haptics on and off delay of PneuClutch, respectively. (b) 35ms and 30ms were observed for haptics on and off delay of PneuIndenter, respectively.

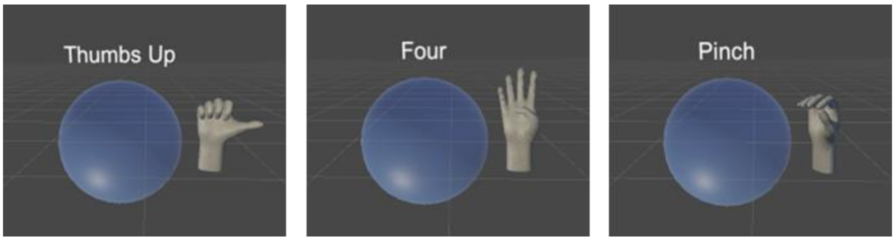

Figure S8. Three-step finger calibration. Users need to do “Thumbs Up”, “Four”, and “Pinch” poeses one by one according to the hand models and touch the blue ball to confirm calibration.

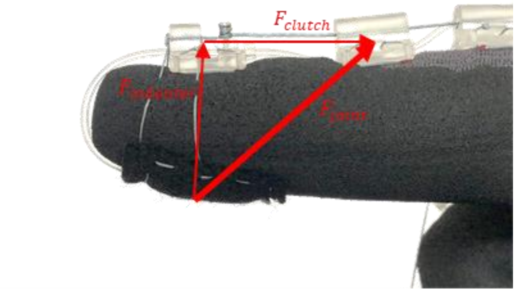

Figure S9. Joint force on fingertip. $\boldsymbol{F_{joint}} \approx \sqrt{\boldsymbol{F_{clutch}}^{2} + \boldsymbol{F_{indenter}}^{2}}$

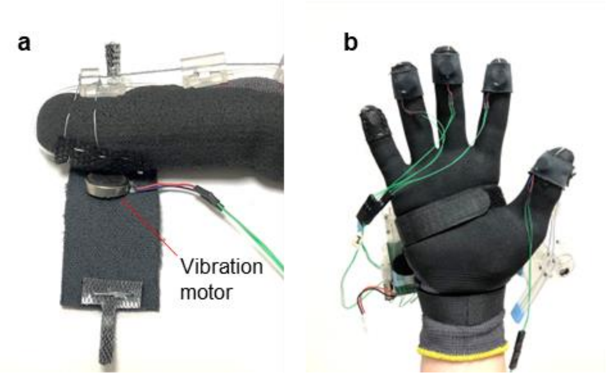

Figure S10. Vibration motors implementation. (a) Vibration motor placed beneath fingertip. (b) Vibration motors implementation for each glove.

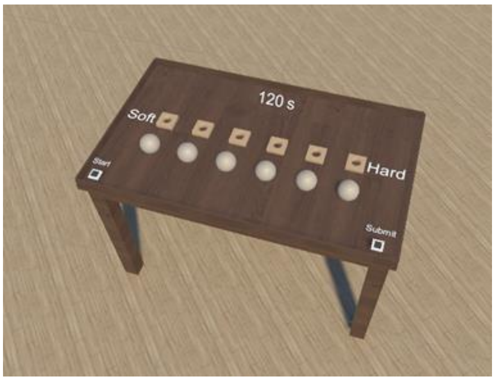

Figure S11. The VR scene of six-ball stiffness sorting.

Table S1. Comparison of commercial and published haptic gloves

Ref.	Kinesthetic feedback				Cutaneous feedback				Finger Tracking	Haptic channels	Weight	Untethered
	**Actuation**	**Variable force**	**Variable stiffness**	**Latency**	**Actuation**	**Variable force**	**Vibration**	**Latency**				
**HaptGlove [a]**												
Our work	Pneumatic	Up to 17.13N	Up to 1.92 N/mm	18ms	Pneumatic	Up to 1.5N	Up to 160Hz	18.8-155.9ms	Stretchable sensors	5	283g	Yes
**Research prototypes**												
[1]	Pneumatic	Up to 4N	Up to 0.092N/mm	130ms	n.a.				n.d.	5	245g [b]	No
[2]	Pneumatic	Up to 3.5N	Up to 0.47N/mm	200-300ms	n.a.				n.a.	5	45.6g [b]	No
[3]	n.a.				Pneumatic	1N	10Hz	200ms	Leapmotion sensor	3	3g/ finger [c]	No
[4]	Motor	Up to 106N	n.a.	21ms	n.a.				Time-of-Flight sensor	4	55g	Yes
[5]	Motor	Up to 3.34N	Up to 0.26N/mm	n.d.	n.a.				Hall sensors	3	488g	Yes
[6]	Electrostatic	Up to 50N	Yes	n.d.	n.a.				Leapmotion sensor	5	28g [d]	No
**Products in the market**												
Meta glove[7]	Pneumatic	n.d.	n.d.	n.d.	Pneumatic	n.d.	n.d.	n.d.	Camera-based tracking	5	n.d.	No
HaptX G1[8]	Pneumatic	Up to 35.59N	n.d.	23ms	Pneumatic	Up to 62kPa	n.d.	n.d.	Magnetic motion tracking	5	450g [b]	No
Dexmo[9]	Motor	Up to 500Nmm	Yes	n.d.	n.a.				Yes	5	300g	Yes
Manus Prime X[10]	n.a.				Vibration	n.a.	Yes	n.d.	Flex sensors and IMU	5	70g	Yes

[a] 0-60kPa actuation pressure

[b] Exclude pneumatic control system

[c] 400g pneumatic control system

[d] Exclude control system

n.d. means not disclosed, n.a. means not applicable

Movie S1.
PneuClutch force feedback with variable actuation pressure.

Movie S2.
PneuIndenter activation with variable pressure and frequency.

Movie S3.
Picking up, manipulating and sensing shape and size of virtual objects.

Movie S4.
Sensing soft and hard objects.

Movie S5.
Sensing the heartbeat, raindrops and engine vibration.

Movie S6.
Pressing a button, and squeezing and bursting a balloon.

Movie S7.
Playing archery.

Movie S8.
Six-ball sorting experiment.

Movie S9.
Socializing with a friend.

References

[1] Y. Zheng, D. Wang, Z. Wang, Y. Zhang, Y. Zhang, W. Xu, Engineering **2018**, 4, 869.

[2] Y. Zhang, D. Wang, Z. Wang, Y. Zhang, J. Xiao, IEEE Trans Haptics **2019**, 12, 269.

[3] G. Frediani, F. Carpi, Sci Rep **2020**, 10, 1.

[4] I. Choi, E. W. Hawkes, D. L. Christensen, C. J. Ploch, S. Follmer, in IEEE International Conference on Intelligent Robots and Systems, IEEE, **2016**, pp. 986–993.

[5] Y. Park, I. Jo, J. Lee, J. Bae, Mech Mach Theory **2020**, 153.

[6] R. J. Hinchet, H. Shea, Advanced Intelligent Systems **2022**, 2200174.

[7] Inside Reality Labs Research: Bringing Touch to the Virtual World | Meta, https://about.fb.com/news/2021/11/reality-labs-haptic-gloves-research/, accessed: 05 **2023**.

[8] HaptX. HaptX Glove., https://haptx.com/, accessed: 05 **2023**.

[9] Dexta Robotics, https://www.dextarobotics.com/, accessed: 05 **2023.**

[10] MANUS Prime X Haptic VR, https://www.manus-meta.com/products/prime-x-haptic, accessed: 05 **2023**.
